# T-1. Assessment of Cytokines and Inflammatory Mediators in Burn Shock Patients After Plasma Inclusive Resuscitation (PIR)

**DOI:** 10.1093/jbcr/irag033.001

**Published:** 2026-04-06

**Authors:** Arthur Kom Sipowa, Amanda Soo Ping Chow, Anthony E Pusateri, Tuan D Le, Thomas Orfeo, Matthew Gissel, Maria Cristina Bravo, Melissa M McLawhorn, Lauren T Moffatt, Jeffrey W Shupp

**Affiliations:** The Burn Center at MedStar Washington Hospital Center, Washington, DC; The Burn Center at MedStar Washington Hospital Center, Washington, DC; Lackland Air Force Base, San Antonio, TX; The Burn Center at MedStar Washington Hospital Center, Washington, DC; Department of Biochemistry, Larner College of Medicine, University of Vermont, Burlington, Vermont; Department of Biochemistry, Larner College of Medicine, University of Vermont, Burlington, Vermont; Department of Pathology and Laboratory Medicine, Larner College of Medicine, University of Vermont, Burlington, VT; MedStar Health, Washington, DC; The Burn Center at MedStar Washington Hospital Center, Washington, DC; MedStar Washington Hospital Center, Washington, DC

## Abstract

**Introduction:**

Severe burns provoke systemic inflammation, leading to endothelial injury and organ dysfunction. In previous work, inflammatory mediators were examined before and after administration of 1 unit of fresh frozen plasma (FFP) in burn patients undergoing plasma inclusive resuscitation (PIR). Modest declines were observed in C1-inhibitor (C1-INH) and TNF-α receptor 1, while IL-6 and soluble IL-6 receptor (sIL-6R) were unchanged. Importantly, a single unit of plasma did not exacerbate inflammation or cause transfusion reactions. We hypothesized that a full PIR course would provide broader anti-inflammatory effects, with biomarkers shifting toward physiologic ranges.

**Methods:**

Adults with ≥20% TBSA burns were prospectively enrolled. PIR was delivered per institutional protocol. Plasma samples were collected at four timepoints: baseline (T0, at admission prior to resuscitation), pre-plasma (T1, immediately before the first unit), post-unit (T2, immediately after the first unit), and completion of PIR (T3, after the last unit). Biomarkers including IL-6, sIL-6R, TNF-α, TNF-α R1/R2, C1-INH, and neutrophil elastase–α1-antitrypsin (NE-AAT) were quantified and reported as medians (IQR). Differences were analyzed with the Friedman test (Prism v10).

**Results:**

Of the 36 patients included, mean age was 43.7 ± 18.4; TBSA 39.1 ± 17.8%; and Baux score 82.8 ± 21.0. Inhalation injury occurred in 27.8% (n = 10), mortality was 25% (n = 9), and median time to first FFP was 186 min (Table-1).

No significant differences were observed among T0–T2 across all markers except C1-INH. C1-INH declined steadily from baseline 198.0% to 130.8% (*p*<.001) at T3. IL-6 was stable at 133–175 pg/mL through T0–T2 but rose sharply at T3 to 708 pg/mL (*p*<.001). sIL-6R declined from 40.3–43.8 ng/mL across T0–T2 to 28.3 ng/mL (*p*<.001) at T3. TNF-α remained stable at 10.1–12.2 pg/mL through T0–T2 but increased at T3 to 14.2 pg/mL (*p*<.001). Both TNF-α receptors rose: TNF-α R1 from 1815–2010 pg/mL (T0–T2) to 2923 (*p*=.003–0.008) at T3, and TNF-α R2 from 3407–3693 pg/mL (T0–T2) to 6113 (*p*<.001) at T3. NE-AAT showed no significant changes (All data, Table 2).

**Conclusions:**

PIR is associated with modulation of inflammatory mediators, with elevations in IL-6, TNF-α, and their receptors, and reductions in sIL-6R and C1-INH. Compared with our prior single-unit PIR study, which showed only modest effects, the full course produced broader modulation without evidence of immune overactivation, supporting PIR’s role in altering the inflammatory trajectory of burn shock. These findings warrant further investigation into PIR potential anti-inflammatory benefits in burn resuscitation.

**Applicability of Research to Practice:**

Cytokine profiling after PIR may help clinicians refine resuscitation strategies to mitigate systemic inflammation and improve outcomes.

**Funding for the Study:**

N/A.

